# Non-coding RNA transcripts and their exosomal counterparts in the pathogenesis, diagnosis, and treatment of skin cancers, with a focus on melanoma

**DOI:** 10.3389/fmolb.2026.1669297

**Published:** 2026-01-29

**Authors:** Linjing Zou, Xuemei Wen, Xiyan Dai, Yaguang Wu

**Affiliations:** The First Affiliated Hospital of Army Military Medical University, Chongqing, China

**Keywords:** skin cancer, melanoma, exosomes, non-coding RNAs, diagnosis, therapeutictargets

## Abstract

Skin malignancies, including melanoma and non-melanoma cancers, are the most common cancers worldwide, with increasing incidence and fatality rates. Malignant melanoma (MM) is a highly aggressive cancer with poor prognosis, and despite various therapies, it remains a serious threat due to factors like tumor recurrence, drug resistance, and lack of effective treatments. Non-coding RNAs (ncRNAs) transcripts have gained attention due to their critical roles in regulating proliferation, angiogenesis, immune regulation, invasion, metastasis, and treatment resistance. Exosomes, biologically active lipid-bilayer extracellular vesicles secreted by various cell types, are also involved in cancer by carrying multiple bioactive molecules, including ncRNAs. Investigating the noncoding components of the transcriptome and their exosomal counterparts opens up the possibility of discovering new therapeutic and diagnostic targets. This review discusses current studies on the involvement of ncRNAs and their exosomal counterparts in the pathogenesis, diagnosis, and treatment of human skin cancers, particularly melanoma.

## Introduction

The evolution of cancer treatments over the past few centuries has seen the introduction of various rationally designed therapies, many of which remain relevant in contemporary oncology practice. Advancements in the understanding of cancer biology have played a crucial role in the progression of cancer therapies. Cancer is fundamentally driven by oncogenic alterations in oncogenes and tumor suppressor genes, which can be somatic or germline in origin. The diversity of tumor types, coupled with the presence of multiple oncogenic alterations and significant intra- and inter-tumor heterogeneity, complicates treatment strategies. These oncogenic changes lead to various effects, including enhanced proliferative signaling, resistance to apoptosis, evasion of replicative limits, and increased genomic instability. The hallmarks of cancer encompass these characteristics and highlight their implications for the development and effectiveness of cancer treatments (Sonkin et al., 2024).

Skin cancers are growths that develop from skin cells and are mainly of two types: non-melanoma skin cancer (NMSC) and melanoma skin cancer. NMSCs arise from epidermal cells and include two main types: basal cell carcinoma (BCC) and cutaneous squamous cell carcinoma (cSCC). BCC is the most common skin cancer, accounting for 75% of NMSC cases, with about 30% of Caucasians likely to develop it during their lives. This type of cancer grows slowly, mainly affecting nearby tissue, and rarely spreads or causes death, with a metastatic rate of less than 0.1%. Key risk factors for BCC include UV light, weakened immune systems, lighter skin, and chronic arsenic exposure. Genetic changes, particularly the inactivation of the PTCH1 gene, play a significant role in BCC development (Cives et al., 2020). cSCC is the second most common skin cancer, making up about 20% of all skin cancer cases. cSCC has a higher risk of spreading and caused 51,900 deaths in 2015. Key risk factors include sunlight exposure and a weakened immune system. cSCC has one of the highest mutation rates, with about 90% of cases showing TP53 inactivation in skin cells, leading to more UV-related mutations. Other affected genes include NOTCH, EGFR, RAS, and CD-KN2A (Caudill et al., 2023).

Melanoma, or malignant melanoma, is a very aggressive and hard-to-treat cancer caused by changes in melanocytes. In 2020, it resulted in around 324,635 new cases and over 57,000 deaths. Despite advancements in treatments such as surgery, chemotherapy, and immunotherapy, the survival rates for melanoma patients remain low. Patients are classified using the American Joint Committee on Cancer criteria, and monitoring for tumor recurrence is essential due to frequent metastasis after initial treatments. The primary cause of melanoma is exposure to ultraviolet (UV) light (Lopes et al., 2022). Common genetic mutations in melanoma include mutant BRAF, RAS, NF1, and Triple-wild-type. About 70% of melanomas have mutations in the MAPK signaling pathway, with BRAF mutations found in around 50% of cases, which contribute to the cancer’s early development. CDKN2A is another significant genetic factor linked to melanoma, affecting vital cell cycle pathways, and changes in this gene occur in 69% of melanoma cases. Based on the data, skin cancers are prevalent globally, and despite progress in their comprehension and treatment, numerous molecular mechanisms remain incompletely investigated. Studies have demonstrated that noncoding RNAs are essential in developmental processes and pathological conditions, with their dysregulation associated with cancer. Noncoding RNAs may serve as valuable biomarkers in the diagnosis and prognosis of cancer outcomes (Guo W. et al., 2021; Motwani and Eccles, 2021). They further offer potential targets for cancer therapy, demonstrating effective nucleic acid-based treatments in preclinical investigations. Exosomes are small vesicles, about 30–150 nm in size, that form from inward budding and are important in both health and disease, including cancer and neurological disorders. They carry various bioactive molecules such as lipids, proteins, and nucleic acids, including different types of ncRNAs. NcRNAs like circular RNAs and microRNAs have significant effects on gene expression. Recent research shows that ncRNA-encoded peptides or proteins can influence tumor growth and development by affecting processes like ubiquitination and metabolism. NcRNAs vary in size and function and are vital in cancer development, immune regulation, and treatment resistance (Huda et al., 2021; Schwarz et al., 2025). Exosomes’ non-coding RNAs (ncRNAs) significantly influence various cellular functions, thereby affecting the field of melanoma research. These functions include the suppression of anti-tumor immune responses, the modulation of drug resistance through miRNA transfer, and the promotion of tumor growth and progression via miRNAs such as miR-222 and miR-155. Additionally, long non-coding RNAs (lncRNAs) like MALAT1 also facilitate metastasis. Furthermore, exosomal ncRNAs can stimulate angiogenesis by enhancing the expression of pro-angiogenic factors in endothelial cells, which aids in melanoma development. Moreover, exosomal ncRNAs can reconstruct tumor microenvironments, further promoting melanoma growth. Overall, exosomal ncRNAs play a crucial role in melanoma treatment and are integral to the understanding of melanoma biology (Marima et al., 2024). Table 1 presents the role of several ncRNAs in melanoma recurrence. Although many studies have looked at ncRNAs in other cancers, their specific function in melanoma is still unclear. This review discusses biogenesis, functions of noncoding RNAs and exosomal ncRNAs, and their potential roles in skin cancer initiation, promotion, and progression.

**TABLE 1:** The regulatory mechanisms of ncRNAs in skin cancers.

ncRNAs name	Molecular function	Expression	Skin cancer subtype	References
MiRNA-204 and miRNA-211	By decreasing the expression levels of the NUAK1/ARK5 protein, miRNA-204 and miRNA-211 increase vemurafenib resistance in melanoma	High expressed	MM	Díaz-Martínez et al. (2018)
miRNA-21	MiRNA-21 facilitates the TIMP3/PI3K/AKT signaling axis, which advances cSCC.	High expressed	cSCC	Yin and Lin (2021)
LINC01291	By boosting the expression of IGF-1R and sponging miRNA-625-5p, LINC01291 promotes aggressive melanoma appearances	High expressed	MM	Wu et al. (2022)
miRNA-203	c-JUN and miRNA-203 control the differentiation and proliferation of basal cells	Low expressed	BCC	Sonkoly et al. (2012)
miRNA-129-5p	In order to carry out its carcinogenic actions, CSDE1 inhibits miRNA-129-5p- in melanoma	Low expressed	MM	Kakumani et al. (2021)
EZR-AS1	Through the PI3K/AKT signaling pathway, EZR-AS1 promotes the migration, invasion, and proliferation of cSCC cells	High expressed	cSCC	Lu et al. (2021)
miRNA-1246	MiRNA-1246 increases the carcinogenicity of melanoma by inhibiting FOXA2	High expressed	MM	Yu et al. (2020)
HOTAIR	By controlling the EMT-related markers Twist, Snail1, and ZEB1 in cSCC, HOTAIR triggers the EMT process	High expressed	cSCC	Yu et al. (2019)
H19	Research on H19’s impact on keratinocyte behavior and the tumor microenvironment is ongoing	High expressed	BCC	Xia et al. (2024)
miRNA-221	By inhibiting PTEN, the oncogenic miRNA-221 accelerates the growth of cSCC.	High expressed	cSCC	Gong et al. (2019)
Circ_0020710	By targeting miRNA-370-3p, Circ_0020710 controls CXCL12, which causes melanoma cells to proliferate, migrate, and invade	High expressed	MM	Wei et al. (2020)
miRNA-143-145 clusters	BCC had downregulated tumor-suppressive cluster miRNA-143-145	Low expressed	BCC	Poli et al. (2020)
Circ_0001591	Circ_0001591 overexpression reduces apoptosis while increasing melanoma cell invasion and proliferation	High expressed	MM	Yin et al. (2021)
miRNA-766	By targeting PDCD5, miRNA-766 increases the tumorigenicity of cSCC.	High expressed	cSCC	Liu et al. (2020)
miRNA-92a	Expression of miRNA-92a is linked to a poor prognosis and tumor stage	High expressed	MM	Sun H et al. (2019)
SPRY4-IT	Although the exact role of SPRY4-IT1 in BCC carcinogenesis is yet unknown, it is thought to influence cell migration, proliferation, and other cellular processes, which in turn contribute to the advancement of cancer	High expressed	BCC	Ghafouri-Fard et al. (2021)
miRNA-451a	In cSCC cells, the tumor suppressor miRNA-451a prevents cell division, migration, invasion, and EMT	Low expressed	cSCC	Fu et al. (2021)
miRNA-494	By suppressing miRNA-494 that is delivered by exosomes, melanoma development and metastasis are prevented	High expressed	MM	Zhang M et al. (2019)
miRNA-3619-5p	By targeting KPNA4, miRNA-3619-5p inhibits the growth of cSCC cells and cisplatin resistance	Low expressed	cSCC	Zhang M et al. (2019)
miRNA-34a	One tumor suppressor that may be employed as a biomarker is miRNA-34a	Low expressed	BCC	Tamas et al. (2023)
miRNA-222	Modulation of melanoma cell plasticity by miRNA-222	High expressed	MM	Lionetti et al. (2020)
CircPVT1	The migration and invasion of cSCC cells are facilitated by the oncogenic circPVT1	High expressed	cSCC	Chen S et al. (2020)
XIST	XIST sponging miRNA-23a-3p and miRNA-217 contributes to the pathogenesis of melanoma to some extent	High expressed	MM	Ma et al. (2019)
Circ-CYP24A1	The exosomal circ-CYP24A1 promotes apoptosis and boosts the migration, invasion, and proliferation of cSCC cells	High expressed	cSCC	Zhang Z et al. (2021)
Circ_0025039	In melanoma, Circ_0025039 enhances glucose metabolism by upregulating CDK4 and inhibiting miRNA-198	High expressed	MM	Bian et al. (2018)
Circ_0070934	Circ_0070934 sponges a number of miRNAs, including miRNA-1236-3p, miRNA-1238, and miRNA-1247-5p, which causes cSCC cells to proliferate and invade	High expressed	cSCC	An et al. (2019)
PRECSIT	PRECSIT alters STAT3 signaling to accelerate the growth of cSCC.	High expressed	cSCC	Piipponen et al. (2020)
miRNA-3662	By targeting ZEB1, ectopic expression of miRNA-3662 suppresses the EMT process and melanoma cell growth	Low expressed	MM	Zhu et al. (2019)
miRNA-199a-5p	Through its targeting of Sirt1 and CD44ICD cleavage signals, miRNA-199a-5p suppresses the stemness of cSCC stem cells	Low expressed	cSCC	Lu et al. (2020)
miRNA-633	By targeting KAI1, the oncogenic miRNA-633 promotes the migration and proliferation of melanoma cells	High expressed	MM	Wang and Liu (2021)
miRNA-675	EMT-related indicators including as vimentin, N-cadherin, and E-cadherin can be impacted by the H19/miRNA-675 axis, which causes EMT.	High expressed	cSCC	Zhang W et al. (2021)
miRNA-200a	Tumor suppressor miRNA-200a modifies the PI3K/Akt signaling pathway and EMT to prevent melanoma cell migration and proliferation	Low expressed	MM	Chen WY et al. (2019)
miRNA-130a	Targeting ACVR1, the tumor suppressor miRNA-130a controls the BMP/SMAD1 pathway	Low expressed	cSCC	Lohcharoenkal et al. (2021)
miRNA-29	Targeting MAFG and MYBL2, the MAPK/miRNA-29 Axis prevents the progression of melanoma	Low expressed	MM	Vera et al. (2021)
Circ_0005795	Circ_0005795 sponging miRNA-1231 stimulates BCC cell growth	High expressed	BCC	Li Y et al. (2021)
miRNA-18a	By suppressing EPHA7 signaling, miRNA-18a-5p promotes the growth of melanoma cells and prevents apoptosis	High expressed	MM	Guo Y et al. (2021)
miRNA-27b	The proliferation of malignant melanoma cells may be influenced by the miRNA-27b/MYC axis	Low expressed	MM	Tian et al. (2021)
miRNA-451a	A key function for the miRNA-451a/TBX1 axis in BCC carcinogenesis	Low expressed	BCC	Sun and Jiang (2018)
miRNA-495-3p	TRAF5 expression and the EMT process are enhanced by HDAC3 through its binding to the miRNA-495-3p promoter	Low expressed	MM	Ma et al. (2020)
MALAT1	The advancement of cSCC is promoted by the MALAT1-KTN1-EGFR axis, which is aided by c-MYC.	High expressed	cSCC	Zhang Y et al. (2019)
Circ_0002770	Through miRNA sponging, Circ_0002770 stimulates melanoma cell invasion and proliferation. -331-3p	High expressed	MM	Zhang et al. (2022)
CASC15	By impacting cell migration, invasion, proliferation, and maybe the EMT pathway, SPRY4-IT1 can increase the tumorigenicity of BCC.	High expressed	BCC	Natarelli et al. (2023)
FUT8-AS1	In individuals with melanoma, FUT8-AS1 downregulation is associated with a worse overall survival rate	Low expressed	MM	Chen XJ et al. (2020)
miRNA-126-3p	By modifying ADAM9 and VEGF-A, downregulation of miRNA-126-3p leads to dabrafenib resistance	Low expressed	MM	Caporali et al. (2019)
Gm26809	Normal fibroblasts are reprogrammed into CAFs by exosomal lncRNA Gm26809	High expressed	MM	Wang D et al. (2022)
miRNA-214	By targeting CADM1, the oncogenic miRNA-214 causes EMT in melanoma	High expressed	MM	Wang SJ et al. (2020)
MIAT	Through the suppression of miRNA-150, MIAT regulates EMT in melanoma	High expressed	MM	Sun X et al. (2019)
TINCR	In nutrient-rich environments, TINCR suppresses invasive melanoma characteristics	Low expressed	MM	Melixetian et al. (2021)
SRA	By activating p38 in melanoma cells, SRA promotes cell invasion, proliferation, and the EMT process	High expressed	MM	Hong et al. (2020)
ZFPM2-AS1	ZFPM2-AS1 stimulates NOTCH1 and sponging miRNA-650 to increase migration and proliferation in melanoma	High expressed	MM	Liu et al. (2021)
CCAT1	In melanoma, CCAT1 stimulates ITGA9 and sponging miRNA-296-3p to promote EMT.	High expressed	MM	Fan et al. (2020)
TSLNC8	The cytotoxic response to the BRAF inhibitor PLX4720 is reduced by TSLNC8 downregulation	Low expressed	MM	Li et al. (2023)
H19	Melanoma cells that express high levels of H19 become resistant to cisplatin by inhibiting miRNA-18b and upregulating IGF1 expression	High expressed	MM	Shi et al. (2018)
Circ-Ccnb1	By dissociating the Ccnb1/Cdk1 complex, Circ-Ccnb1 reduces the migration, proliferation, and survival of melanoma cells	Low expressed	MM	Fang et al. (2019)

### NcRNAs in cancers

**FIGURE 1:**
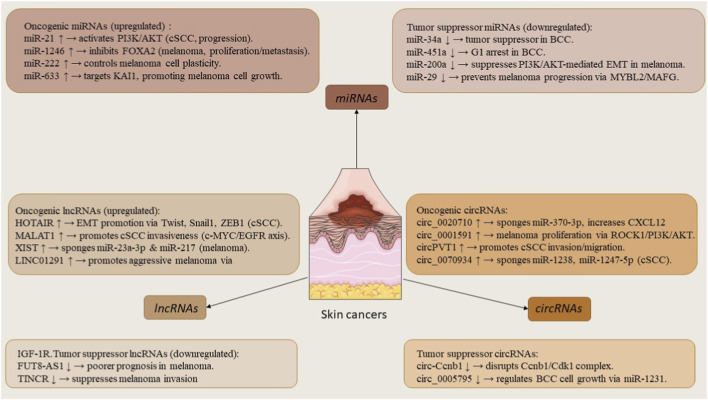
Overview of dysregulated microRNAs, long non-coding RNAs, and circular RNAs in basal cell carcinoma, cutaneous squamous cell carcinoma, and melanoma. These ncRNAs act as oncogenes or tumor suppressors, regulating hallmarks such as proliferation, apoptosis, EMT, angiogenesis, metastasis, and drug resistance.

NcRNAs do not encode proteins but regulate gene expression through various mechanisms. Over the past 30 years, they have gained recognition as important regulators in normal cell functions and diseases like cancer. They are divided into short and long types based on a 200-nucleotide length. They play roles in many cellular functions like gene transcription, regulation, and chromosome stability. Circular RNAs (circRNAs) are a new type of RNA with unclear functions but may be promising biomarkers in human cancers. Noncoding RNAs play important roles in cancer by helping cells avoid death, grow blood vessels, continue growing, and resist drugs. They can act as tumor suppressors or oncogenes and are often misrelated in cancer. The next sections will discuss key noncoding RNAs involved in skin cancer (Figure 1) (Kaikkonen et al., 2011).

### MicroRNAs (miRNAs)

MiRNAs, are small non-coding RNAs about 19–25 nucleotides long. They can target multiple genes simultaneously and play important roles in biological processes. Most miRNA genes are made in the nucleus by RNA polymerase II and III. They attach to specific sequences at the 3′UTR of target mRNAs, causing translational repression and mRNA deadenylation and decapping. Profiling miRNAs is important for diagnosing and predicting the outcome of various cancers, including skin cancers, and some miRNAs may even serve as therapeutic targets (O'Brien et al., 2018).

### Functional role of miRNAs in skin cancers

In the last decade, many studies have focused on the role of miRNAs in skin cancers, showing interest in their impact on cancer initiation, development, and spread. MiRNAs can regulate gene expression, and changes in one can affect many target mRNAs. Several studies have noted abnormal miRNA expressions in BCC. The expression of miRNA-451a is significantly lower in human and mouse BCC tissues. When miRNA-451a is overexpressed in BCC cells, it slows cell growth by causing G1 cell cycle arrest. On the other hand, reducing miRNA-451a increases BCC cell growth and colony formation, highlighting its role as a tumor suppressor. Similarly, miRNA-34a levels are lower in BCC patients compared to healthy individuals and are linked to poorer prognosis. MiRNA-203, mainly found in skin tissue, is also downregulated in BCC due to activation of the Hedgehog pathway and the EGFR/MEK/ERK/JUN signaling pathway. Finally, miRNA-145-5p is significantly reduced in BCC (Sun and Jiang, 2018; Abd-Allah et al., 2023).

MiRNA-21 is a well-known cancer-related microRNA that is increased in many cancers. It promotes disease progression in cutaneous squamous cell carcinoma (cSCC) by affecting the PI3K/AKT pathway through TIMP3 regulation. Inhibition of miRNA-21 in mouse models decreased tumor growth and spread. Silencing TIMP3 reversed the effects of miRNA-21 downregulation (Yin and Lin, 2021). Additionally, miRNA-130a acts as a tumor suppressor by regulating the BMP/SMAD1 pathway in cSCC. Overexpression of miRNA-130a reduces long-term growth, cell movement, and invasion ability. Cancer stem cells (CSCs) can start new tumors and cause relapses, identified mainly through markers like CD44, CD24, CD166, and CD133. It has been found that miRNA-199a-5p affects CD44 proteolysis, leading to decreased cell growth and reduced stemness in cSCC CSCs. This is due to miRNA-199a-5p preventing the breakdown of CD44 and lowering its nuclear movement by targeting Sirt-1, which boosts the expression of Oct4, Sox2, and Nanog. Additionally, miRNA-766 promotes cell growth and movement in cSCC, while miRNA-221 drives tumor growth by inhibiting the suppressor gene PTEN (Lohcharoenkal et al., 2021; Wang et al., 2014).

Numerous studies show that miRNAs are expressed abnormally in melanoma. The miRNA-29 family, which includes miRNA-29a, miRNA-29b, and miRNA-29c, targets the oncogenes MYBL2 and MAFG, promoting cell growth in melanoma. Lowering miRNA-29b2c levels can lead to melanoma development by activating these oncogenes. The KAI1 gene, a key tumor suppressor, is targeted by miRNA-633, which boosts melanoma cell growth and movement. Additionally, miRNA-18a is highly present in melanoma and enhances cell growth while reducing cell death by suppressing EPHA7 expression (Vera et al., 2021; Li L. et al., 2021).

Myc, a family of proto-oncogenes, plays a crucial role in tumorigenicity and regulates over 15% of the genome. MiRNA-27b, a miRNA, can inhibit melanoma progression by targeting MYC. Its expression levels in melanoma tissue samples are lower than normal tissues, and ectopic overexpression significantly decreases melanoma cell DNA synthesis, vitality, and invasive ability. Melanoma cell plasticity, a key factor in cancer spread, is influenced by cellular plasticity. Recent research has shown that miRNA-222 is a key factor controlling melanoma plasticity, a molecular and phenotypic change that cancer cells exhibit as it progresses (Frenzel et al., 2010). FOX proteins, a family of transcription factors, are mutated in various human cancers. MiRNAs can regulate FOX proteins in melanoma, such as miRNA-1246, miRNA-92a, and miRNA-182. MiRNA-1246 promotes melanoma cell viability and metastasis by suppressing FOXA2, miRNA-92a is upregulated in melanoma and linked to tumor stage and poor prognosis. MiRNA-182 targets FOXO3 and promotes metastasis. CSDE1, an oncogenic RNA-binding protein, promotes tumorigenicity in various cancers. In melanoma, CSDE1 and AGO2 compete to bind PMEPA1 mRNA, leading to upregulation of PMEPA1 and inhibiting miRNA-129-5p-mediated silencing. The study suggests that miRNAs can play a dual role in skin cancer pathogenesis, and a better understanding of their roles in malignancy initiation and development can lead to better translation of miRNAs into clinics for skin cancer treatment (Yu et al., 2020; Bouzari et al., 2022).

The mitotic DNA integrity checkpoint signaling pathway is linked to cancer and genomic stability, involving 16 key protein kinase genes: ATM, BRSK1, CDK1, CDK2, CHEK1, CHEK2, MAP3K20, NEK11, PLK1, PLK2, PLK3, PRKDC, STK33, TAOK1, TAOK2, and TAOK3. This study aims to create profiles of these genes across different cancers for potential use in diagnosis, prognosis, and therapy. Researchers gathered multi-omic data from over 9,000 samples across 33 cancer types, analyzing mutations, copy number variations, methylation, mRNA expression, and microRNA networks. Findings revealed high mutation frequencies for these genes in certain cancers like UCEC and SKCM, while CNVs were linked to survival in UCEC, KIRP, and LGG. The mRNA expression of these genes could affect BRCA, KIRC, LUAD, and STAD. These genes also interact with other cancer pathways. Overall, this research highlights the potential of protein kinases as biomarkers for cancer, although further validation is needed before clinical applications can be developed (Rasteh et al., 2024; Li et al., 2025). A study investigates how miRNA expression affects resistance to cancer treatments. miRNAs, which help control gene expression in cancer cells, are linked to treatment failure. Drug resistance remains a major issue for patients with advanced cancer, particularly resistance caused by miRNAs that target drug-related genes. In resistant cells, certain pathways are suppressed, leading to increased resistance. Understanding these miRNAs is essential for improving drug selection and combination therapies. Gaining insights into their role in drug resistance can enhance treatment options and improve patient outcomes (Li et al., 2025; Liu and Tang, 2023).

### Long noncoding RNAs (Lnc RNAs)

Long noncoding RNAs, or lnc-RNAs, are RNA transcripts longer than 200 nucleotides that are not translated into protein. They are more abundant than microRNAs but less conserved through evolution. The exact number of functional lnc-RNAs is still debated, yet many have important roles in cellular functions. Abnormal lncRNA expression is linked to several human diseases, including cancer. Lnc-RNAs help regulate genes, impacting gene expression in various ways. Their improper regulation can lead to human diseases, including skin cancers (Mattick et al., 2023).

The research investigates the role of FOXD2-AS1 in the development of oral squamous-cell carcinoma (OSCC), particularly in cell invasion and migration. Scientists used various techniques, including prognostic and bioinformatics analyses, to study the expression levels of FOXD2-AS1 and its relationship with the PLOD1 gene. They altered the levels of FOXD2-AS1 in cancer cells and measured changes in cell viability, migration, and invasion. The study confirmed that FOXD2-AS1 is highly expressed in OSCC and is linked to cancer prognosis. Reducing FOXD2-AS1 levels decreased cell activity and movement, as well as affected gene expression related to these processes. Inhibition of FOXD2-AS1 slowed tumor growth in animal models, showing that it negatively regulates miR-185-5 p, which in turn affects PLOD1. The study found that FOXD2-AS1 and PLOD1 are linked to the Akt/mTOR signaling pathway, suggesting that increased FOXD2-AS1 promotes OSCC growth and invasion through this pathway (Liu J. et al., 2024).

Gastric cancer (GC) is the third leading cause of cancer-related deaths worldwide, primarily due to challenges associated with late diagnosis and limited treatment options. lncRNAs have emerged as promising targets for improving cancer prognosis, diagnosis, and therapy. Their high specificity and the ability for non-invasive detection in body fluids make them particularly valuable in clinical settings. Research highlights the significant role of lncRNAs in various stages of GC pathogenesis, including initiation, metastasis, and recurrence, suggesting their potential as novel biomarkers for diagnosis and prognosis, as well as therapeutic targets. Despite the potential of lncRNAs, there are still hurdles to their clinical application in gastric cancer. However, advancements in the understanding of lncRNA molecular biology offer hope for enhancing treatment outcomes and survival rates for patients with GC. This review synthesizes recent findings on lncRNAs in gastric cancer, detailing their molecular mechanisms and discussing their prospective clinical applications, thereby underscoring the importance of continued research in this area (Ghorbani et al., 2024).

### The functional role of lncRNAs in skin cancer carcinogenesis

lncRNAs are important in skin cancers as they help control cell growth, death, blood vessel formation, invasion, and the characteristics of stem cells. Research suggests lncRNAs could also affect the skin tumor environment and metastasis. There is limited information on lncRNAs in BCC, and more studies are needed to understand their role in BCC. Some lncRNAs, like H19, CASC15, and SPRY4-IT, are upregulated in BCC, but their functions require further exploration. In cSCC, the lncRNA PICSAR is highly expressed and might be a potential biomarker. Reducing PICSAR levels can hinder cell growth and invasion, suggesting new treatment options for cSCC (Chen et al., 2017). The H19/miRNA-675 axis plays a role in the development, spread, and advancement of cSCC. LINC00346, or PRECSIT, is another lncRNA that boosts cSCC cell invasion by activating STAT3 and lowering certain protein levels. EZR-AS1, found on chromosome 6q25.3, increases cell movement and cancer differentiation; its reduction decreases cSCC cell growth and spread while encouraging cell death through the PI3K/AKT pathway (Zhang W. et al., 2021). Additionally, MALAT1, influenced by UVB exposure, is linked to increased cSCC cell growth and invasiveness while reducing cell death. MALAT1 promotes cancer by connecting with c-MYC and binding to the KTN1 gene’s promoter, which helps increase EGFR protein levels. The roles of lncRNAs in melanoma are being studied. XIST, a lncRNA on the X chromosome, is highly expressed in melanoma cells and aids disease progression by sponging miRNA-23a-3p and targeting GINS2. Additionally, XIST helps melanoma metastasis by sponging miRNA-217. Analysis from The Cancer Genome Atlas shows that FUT8-AS1 may relate to melanoma prognosis; it is less active in melanomas than benign nevi, leading to poorer survival. FUT8-AS1 works as a tumor suppressor and controls cell behavior by influencing miRNA-145-5p and downregulating NRAS, which suppresses MAPK signaling (Wang Y. et al., 2020; Juárez-Vicuña et al., 2024). The lnc-RNA ZFPM2-AS1 increases melanoma cell growth and movement by sponging miRNA-650 and activating NOTCH1. LINC01291 also promotes aggressive melanoma by sponging miRNA-625-5p, raising IGF-1R levels. In stressed melanoma cells, ATF4 controls responses, while in nutrient-rich conditions, lncRNA TINCR reduces invasive traits by inhibiting ATF4 translation. Immunotherapy for melanoma is improving, aiming to enhance patient survival. A study identified 15 lncRNAs that may predict survival benefits from anti-PD-1 treatment. LncRNAs such as NARF-AS1 and LINC01126 are differently expressed in training and validation groups and are linked to immune processes and treatment. There is growing evidence that lncRNAs play an important role in skin cancer development. Evaluating their use could help in diagnosing and treating skin cancers (Wu et al., 2022).

### Circular RNAs (circRNAs)

CircRNAs are important because they play various roles in cells that can affect traits and diseases. They can change how genes are expressed or how proteins are made by acting as decoys for miRNAs or RBPs. Recent studies also suggest that circRNAs might be useful as biomarkers for human cancers. CircRNAs form through back-splicing, linking the ends of exons to create a closed structure, which makes them more stable than linear RNAs, protecting them from breakdown. While they were once seen only as noncoding RNAs with regulatory roles, it has been demonstrated that they can also be translated into proteins. CircRNAs use different mechanisms to function, including acting as sponges for miRNAs, binding proteins, and regulating gene expression (Gu et al., 2023).

### The epigenetic role of ncRNAs in skin cancers

CircRNAs are important in the development of skin cancers. A study identified 48 downregulated and 23 upregulated circRNAs in BCC. Circ_0005795 is higher in BCC tissues and cells, serving as a potential biomarker and promoting cell growth by targeting miRNA-1231. A recent study found that 449 circRNAs have different levels in cSCC compared to normal tissue. CircPVT1 is one such circRNA that, when reduced, stops cell movement and invasion. Another circRNA, Circ_0070934, is also higher in cSCC and supports cancer growth by binding to miRNA-1238 and miRNA-1247-5p (Liu and Li, 2022). Circ-0070934 regulates HOXB7, which is linked to cancer development. Reducing Circ-0070934 led to less invasive and proliferative activity in cSCC cells and increased cell death. Several studies show that the CXCL chemokine family is important in human skin cancers, particularly melanoma. The circular RNA circ_0020710 boosts CXCL12 levels by targeting miRNA-370-3p, which promotes melanoma cell growth and spread. The proteins Ccnb1 and Cdk1 work together in various cancers, and circ-Ccnb1 disrupts their interaction, reducing melanoma cell migration and survival. Another circular RNA, circ_0001591, is found in higher amounts in melanoma patients, leading to increased cell growth and reduced cell death by activating specific proteins through miRNA-431-5p repression. Melanoma cells mainly use glycolysis for energy. Circ_0025039 enhances glucose metabolism in melanoma cells by affecting miRNA-198 and raising CDK4 levels. Also, circ_0002770, linked to the oncogene MDM2, promotes melanoma cell growth and invasion. CircRNAs are important in the progression of skin cancers. Their stable, closed-loop structure makes them more enduring than other noncoding RNAs, influencing various biological processes. They are also considered good biomarkers for liquid biopsies due to their unique qualities (Fania et al., 2021; Zambrano-Roman et al., 2022).

### The role of ncRNAs in skin cancers’ epithelial-mesenchymal transition mechanism

**FIGURE 2:**
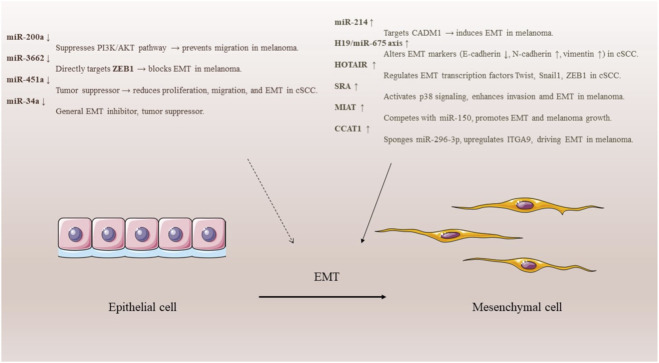
Non-coding RNAs regulating the epithelial-mesenchymal transition (EMT) in skin cancers. Oncogenic ncRNAs promote EMT via pathways such as CADM1, PI3K/AKT, and EMT transcription factors, while tumor-suppressive ncRNAs inhibit this process.

The epithelial-mesenchymal transition (EMT) is an important process in cancer spread where epithelial cells gain mesenchymal traits. EMT plays a role in early development and tissue healing, but it also contributes to cancer growth and treatment resistance. Various transcription factors control EMT, and noncoding RNAs may regulate these factors in different diseases, (Figure 2). In cSCC cells, the H19/miRNA-675 axis influences EMT markers like E-cadherin and vimentin, promoting EMT. miRNA-451a acts as a tumor suppressor, and its expression slows down cell growth and movement while increasing cell death. HOTAIR also promotes EMT by regulating markers such as Twist and Snail1. CADM1 is a gene that may stop the EMT process. A study found that miRNA-214 causes EMT in melanoma by targeting CADM1. miRNA-200a can reduce melanoma cell growth and movement by affecting the PI3K/Akt pathway and EMT. ZEB1 is a main regulator of EMT, and miRNA-3662 can inhibit EMT and melanoma growth by targeting ZEB1 (Zhang W. et al., 2021; Zhang and Weinberg, 2018). Research also showed that lower levels of miRNA-495-3p and higher levels of HDAC3 occur in melanoma, with HDAC3 affecting miRNA-495-3p expression. LncRNA SRA is noted for its cancer-causing roles in breast and prostate cancers. In melanoma, SRA helps the EMT process, increases cell invasion, and promotes growth by activating p38. The lncRNA MIAT also regulates EMT by competing with mi-RNA-150, enhancing growth and invasion. Similarly, lncRNA CCAT1 supports EMT by sponging miRNA-296-3p and increasing ITGA9 levels. This shows the important role of noncoding RNAs in EMT regulation and potential targets for skin cancer treatment (Figure 2) (Ma et al., 2020).

## NcRNAs in skin malignancies resistant to drugs

**FIGURE 3:**
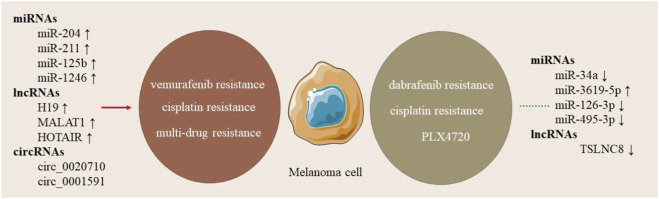
ncRNAs influence drug resistance in melanoma and other skin cancers. Oncogenic ncRNAs (red) drive resistance to BRAF inhibitors and chemotherapy, whereas tumor-suppressive ncRNAs (green) restore sensitivity through apoptosis regulation, signaling control, and drug-response pathways.

Drug resistance is a major challenge in treating skin cancers. Noncoding RNAs play a role in either supporting or fighting this resistance (Figure 3). They can influence drug resistance through various mechanisms, including the regulation of drug efflux pumps, evasion of apoptosis, modification of the cell cycle, promotion of epithelial-to-mesenchymal transition (EMT), and alteration of drug metabolism. These processes can result in heightened invasiveness and resistance in cancer cells, along with changes in drug availability and efficacy. Furthermore, ncRNAs have the capacity to inhibit pro-apoptotic genes or upregulate anti-apoptotic genes, thus improving the survival and resistance of cancer cells. MicroRNAs play a crucial role in drug resistance in melanoma, with downregulation of miR-211 linked to increased resistance to BRAF inhibitors. MiR-34a, known for regulating apoptosis and cell cycle, is also associated with resistance to chemotherapeutic agents. Overexpression of miR-125b has been linked to resistance to vemurafenib, a BRAF inhibitor. Long Non-Coding RNAs (lncRNAs) like HOTAIR, MALAT1, and UCA1 contribute to drug resistance by regulating chromatin states and gene expression. Circular RNAs act as sponges for miRNAs that regulate drug resistance genes, sequestering miRNAs that suppress oncogenic pathways, contributing to drug resistance in melanoma. However, specific circRNAs are still being extensively studied in skin malignancies. In cSCC, miRNA-3619-5p helps block resistance to cisplatin by targeting KPNA4, which is linked to cancer growth. LncRNA PICSAR is also involved in resistance by affecting miRNA-485-5p and increasing REV3L levels. In melanoma cells, miRNA-126-3p is less active and helps cause resistance to dabrafenib by affecting ADAM9 and VEGF-A. MiRNA-204 and miRNA-211, which are similar, promote vemurafenib resistance by lowering NUAK1/ARK5 protein levels. Additionally, the lncRNA H19 is linked to cisplatin resistance by sponging miRNA-18b and raising IGF1 expression. TSLNC8 is lower in BRAF inhibitor-resistant cells, which weakens the response to the inhibitor PLX4720 (Zhang M. et al., 2019; Wang D. et al., 2020).

## Characterization and isolation of exosomes

### Isolation of exosomes

Exosomes are small vesicles with a bilayer membrane, first described in 1983. They have a cup-shaped structure and range from 30 to 150 nm in size. Exosomes are small vesicles released from cells that carry proteins, lipids, and genetic material, including noncoding RNAs. The isolation of exosomes plays a crucial role in studying non-coding RNAs (ncRNAs) due to their function as protective carriers for these molecules between cells. Exosomes encapsulate various forms of ncRNAs, including microRNAs (miRNAs), long non-coding RNAs (lncRNAs), and circular RNAs (circRNAs), safeguarding them from enzymatic degradation present in bodily fluids. Therefore, isolating exosomes is a vital step in obtaining stable and viable ncRNAs needed for thorough analysis. The purity and specificity of the isolation process significantly affect the quality of the ncRNA yield extracted from exosomes. Contaminants such as free-circulating RNAs and proteins can interfere with research findings, emphasizing the need for effective isolation methodologies (Lin et al., 2020). Each method has its advantages and disadvantages; for instance, ultracentrifugation is extensively utilized for its ability to yield relatively pure exosomes, ensuring that the ncRNAs extracted are indeed exosomal. In contrast, size exclusion chromatography and immunoaffinity capture offer higher purity, which is beneficial for enhancing the specificity of ncRNA studies. On the other hand, precipitation kits provide ease of use, but they may inadvertently co-isolate non-exosomal RNA, which could compromise the accuracy of subsequent ncRNA analyses. Once the exosomes have been isolated, specialized methods for RNA extraction are implemented to separate ncRNAs. This extracted RNA can further be analyzed using techniques such as quantitative PCR (qPCR), RNA sequencing, or microarrays. These analytical methods facilitate a deeper understanding of the expression profiles and biological functions of ncRNAs, establishing their potential roles as biomarkers in various diseases. In summary, efficient and pure exosome isolation is essential for the study of encapsulated ncRNAs, as this directly influences the validity of research findings regarding ncRNAs and their potential implications in disease identification and treatment (Konoshenko et al., 2018).

Understanding how exosomes form and specify their contents is important for their potential use as biomarkers and therapies. Recent studies show that tumor cells utilize exosomes to share harmful noncoding RNAs with each other and with normal cells (Lin et al., 2020). Several techniques are used to purify exosomes, with ultracentrifugation being the most common, accounting for 56% of separations. Shu et al. created a quick method for isolating exosomes from melanoma cell cultures using ultrafiltration and size exclusion chromatography, confirming their results with molecular and microscopic analyses. Typical detection methods include Nanoparticle Tracking Analysis, Western blot, and Transmission Electron Microscopy. A new method called Flow Field-Flow Fractionation has been introduced, along with dual mode chromatography combined with Size Exclusion Chromatography to improve exosome purity (Li et al., 2017). An Deun et al. used DMC to successfully reduce lipoprotein particles in plasma exosome preparations. Although ultrafiltration is fast and does not need costly machines, it does not effectively enrich exosomes. Other methods like immune affinity capture isolate exosomes based on protein interactions but yield less. Recently, several kits for exosome enrichment have been released, including ExoQuick, qEVcolumns, Amicon® ultra filters, and exoEasy Maxi kit (Ter-Ovanesyan et al., 2023). Guo et al. noted that microfluidics-based systems for isolating and analyzing extracellular vesicles may help in AI-based disease diagnosis (Guo et al., 2018).

### Characterization of exosome

Exosomes are small extracellular vesicles that serve as key vehicles for transporting various molecular components, with ncRNAs playing a critical role in their biological functions. The characterization of exosomes, particularly in regard to their ncRNA content, involves understanding both their physical attributes and the array of RNA molecules they carry. On the molecular side, profiling the ncRNA within exosomes begins with RNA extraction. This process requires specialized protocols to minimize contamination from free-floating RNA. Following extraction, RNA quantification and size distribution analysis are conducted using bioanalyzers or similar platforms, which generally show a significant presence of small RNA species (Gan et al., 2025). The identification of different types of ncRNAs, including microRNAs (miRNAs), long non-coding RNAs (lncRNAs), circular RNAs (circRNAs), as well as smaller RNAs such as snoRNAs and piRNAs, is achieved through high-throughput RNA sequencing (RNA-seq), microarrays, or qRT-PCR. In addition to physical and molecular characterization, understanding the functional implications of ncRNAs associated with exosomes is crucial. Investigating how these ncRNAs influence recipient cells can affirm their biological significance. Functional assays might include gene expression analysis, phenotypic assessment, and pathway studies. Characterizing exosomal ncRNAs offers significant benefits, such as enabling the discovery of biomarkers which are uniquely stable in bodily fluids, thereby providing insights into the physiological or pathological states of their cells of origin. This characterization is fundamental for grasping how exosomal ncRNAs facilitate intercellular communication and exert their influences (Liu S. et al., 2024).

**FIGURE 4:**
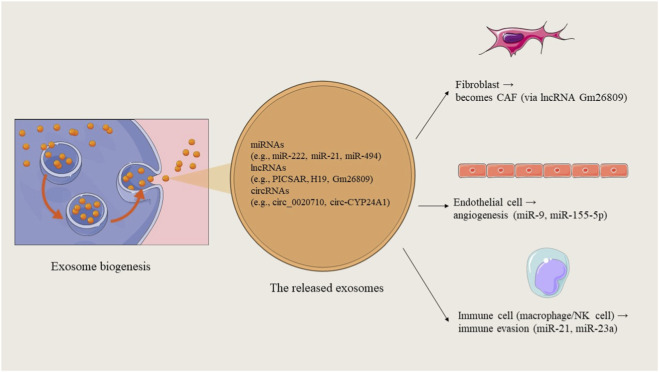
Biogenesis of melanoma-derived exosomes and their cargo. Exosomes form via the endosomal pathway, become enriched in ncRNAs, proteins, and lipids, and are released into the tumor microenvironment to interact with recipient cells.

Exosomes play important roles in monitoring melanoma metastasis and facilitating communication between cells, impacting tumor growth. Many studies are now focusing on exosomes because they contain various cellular components, such as proteins, lipids, and RNA (Figure 4). They have unique features like size, shape, and specific marker proteins that help identify them. Characterization of exosomes is usually done using techniques like Western blot, but their changing nature makes it hard to recognize specific markers during their development. Other methods for characterizing exosomes include NTA, TEM, Flow Cytometry, and Atomic Force Microscopy. Western blot is a technique often used to measure specific proteins like CD9, CD63, and CD81 in exosomes. When identifying cancer biomarkers, it is important to consider certain proteins that are usually found in exosomes from melanoma cells, such as PD-L1 (Gowda et al., 2020). Arraud and others studied exosome characteristics in plasma using cryo-TEM and gold labeling, finding concentrations between 10^4 and 10^12 per milliliter. NTA is a quick and simple method that allows samples to return to their original form after testing. Kashkanova introduced iNTA, a new method for better sensitivity and accuracy in analyzing exosomes and their diversity. Flow Cytometry is a useful method for analyzing clinical samples repeatedly (Hartjes et al., 2019). Inglis et al. introduced two protocols for isolating and analyzing Extracellular Vesicles (EVs). One protocol works well for many clinical samples, while the other, bead-based method is better for finding and capturing exosomes. Tiruvayipati et al. used Tunable Resistive Pulse Sensing (TRPS), which offers precise detection of exosomes. Atomic Force Microscopy can examine exosomes in their natural state without needing sample preparation. Sung et al. developed improved tools for better detection of exosome secretion in cells. However, the isolation and analysis of exosomes still face challenges due to inefficient procedures, storage problems, and a lack of quality controls (Konoshenko et al., 2018).

### The biological function of exosomal ncRNAs in melanoma

The tumor microenvironment (TME) in cancer is known to be immunosuppressive and contains various cell types within an extracellular matrix. Cancer-associated fibroblasts (CAFs) are a key component, contributing to cancer growth and resistance to treatments. Extracellular vesicles (EVs), which include exosomes and microvesicles, are important biological nanoparticles that carry various cellular materials like proteins and miRNAs. These EVs facilitate communication between CAFs and tumor cells, with those from tumor cells activating fibroblasts and those from CAFs promoting breast cancer progression (Li et al., 2024). Actually, these EVs help transfer important molecules like ncRNAs, proteins, carbohydrates, and enzymes between tumor cells and other cells, playing a crucial role in melanoma development and growth. Recent studies show that miRNAs in exosomes can influence melanoma cell creation, spread, and invasion. Specifically, miR-191 and miR-425 promote invasion and growth, while miR-206 acts in the opposite way, with its effects being reduced by estrogens. Table 2 outlines the potential roles of exosomal ncRNAs in melanoma.

**TABLE 2:** Possible exosomal ncRNA role in melanoma.

ncRNAs	Molecular and signal mechanism	References
miR-222	Increase the malignancy of melanoma tumors PI3K/AKT signaling upstream and via the RAS/MAPK	Felicetti et al. (2016)
miR-155-5p	Use the SOCS1/JAK2/STAT3 signaling pathway to cause cancer-associated fibroblasts to undergo a proangiogenic switch	Zhou et al. (2018)
hsa_circ_0001005	Use the regulatory ceRNA network to increase melanoma’s resistance to vemurafenib	Wang and Cheng (2023)
miR-4535	Facilitate the spread of melanoma by preventing the autophagy process from	Liu et al. (2023)
miR-1268a	By inhibiting the autophagy mechanism, melanoma invasion and migration can be promoted	Li et al. (2022)
miR-138-5p/SOX4	Boost the apoptosis and tumor proliferation through the control of SOX4 transcription	Wang X et al. (2022)
miR-411-5p	Increase the spread of melanoma through the MAPK/ERK signaling pathway’s activation	Chen H et al. (2022)
miR-22-3p	Prevent the melanoma cells’ epithelial-mesenchymal divide through controlling LGALS1	Chen Y et al. (2022)
lncRNA-Gm26809	Increase the migration and proliferation of melanoma cells by reprogramming fibroblasts to produce tumor-promoting via transferring Gm26809 using CAFs via	Hu and Hu (2019)
miR-211-5p	control melanoma’s immunological milieu, pyroptosis, and glucose metabolism by controlling GNA15	Zeng et al. (2023)
circular RNA circ_ 0020710	propels immunological evasion and tumor growth through controlling the melanoma’s miR-370-3p/CXCL12 axis	Wei et al. (2020)
miR-122-5p	Facilitate the survival and multiplication of melanomaLPAR3 SH3-binding domain to control Wnt1	Byrnes et al. (2019)
miR-494	Increase the melanoma growth and spread through controlling Bcl-2, an antiapoptotic protein,	Wacheck et al. (2003)
miR-2478	inhibits melanogenesis through the Akt-GSK3β pathway	Bae and Kim (2021)
miR-199a-1-5p	Boost the capacity to spread Targeting OL cells increases metastatic colonization by deactivating the cell cycle inhibitor CDKN1B	Zhao et al. (2022)
miR-21	Control the genesis of melanoma by inhibiting the anti-apoptotic BCL2 protein found in the mitochondrial membrane	Melnik (2015)
miR-106b-5p	Targeting EphA4 will help primary melanocytes undergo the epithelial-mesenchymal transition	Luan et al. (2021)
miR-300	Control the growth of melanoma through the TFs-mRNA-miRNA axis	Chen L et al. (2019)

**FIGURE 5:**
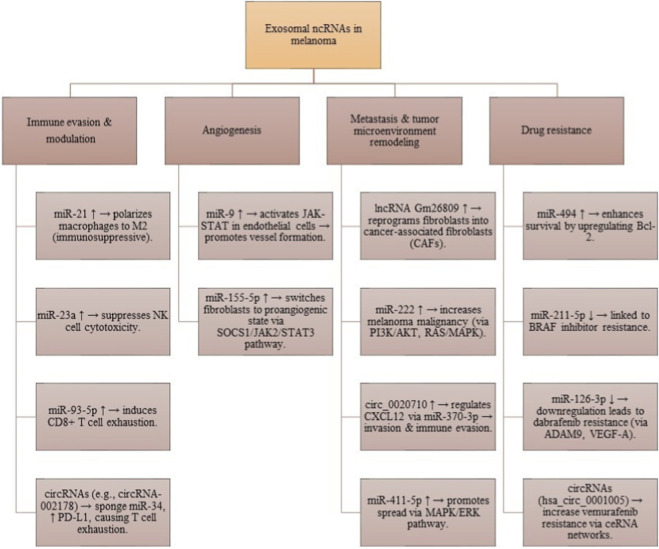
Functional roles of exosomal ncRNAs in melanoma. These molecules contribute to immune evasion, angiogenesis, fibroblast reprogramming, metastasis, and therapy resistance, underscoring their importance as biomarkers and therapeutic targets.

Let-7a helps tumor cells invade by increasing E-cadherin and decreasing vimentin through interactions with LIB28B and HMGA2. Another study by the Felicetti group looked at exosomes using Exoquick-TC® or ultracentrifugation to explore the role of miR-222 in melanoma, suggesting involvement of the PI3K/AKT pathway with the BKM120 inhibitor. This indicates that exosomal miRNAs could be useful as biomarkers and therapies for melanoma (De Sousa et al., 2023). Que and colleagues found that miRNA-depleted exosomes from tumor cell supernatants activate DC/CIK. Additionally, exosomes transport miR-125b-5p to macrophages, influencing their behavior to support tumor growth. Li et al. found that exosomes from aggressive melanoma cancer stem cells speed up the spread of melanoma by making less aggressive melanoma cells more invasive. Targeted therapies for melanoma often fail due to drug resistance. Some exosomal miRNAs can cause resistance to treatments, while others can help overcome it. An *in vitro* study by Sun et al. showed that miR-7 can inhibit resistance to vemurafenib in melanoma. MiRNAs can change the expression of various targets, sometimes acting as cancer-promoters or suppressors in different contexts. Exosomal lncRNAs also play key roles in regulating gene expression and the cell cycle in melanoma, affecting tumor growth and spread. For example, the lncRNA PVT1 helps melanoma grow by promoting cancer cell activity and reducing PTEN expression (Que et al., 2016; Balaraman et al., 2025). Certain lncRNAs can even prevent the growth and movement of melanoma cells, hindering cancer progression. HOTAIR is a lncRNA that promotes melanoma by helping cancer cells move and invade, increasing the chance of tumor spread. Research by Wu et al. showed that the lncRNA MEG3 also encourages melanoma growth and spread, linking it positively to miR-21 and negatively to E-cadherin. LncRNAs can affect processes like EMT, with UCA1 activating EMT during melanoma. LncRNAs can regulate genes by changing chromatin structure or protein function. Additionally, exosomal circRNAs like circ-SHKBP1 aid in melanoma growth and maintenance. Recent studies show that exosomes carry circRNAs to other cells, affecting various biological processes. For instance, exosomal circ-FARSA leads to the breakdown and modification of the PTEN protein in macrophages, impacting the PI3K/AKT pathway. Exosomal circ-0048117 in esophageal squamous cell carcinoma (ESCC) acts as a sponge for miR-40, influencing its function and promoting M2 polarization in macrophages. Additionally, has-circRNA-002178 can boost PD-L1 expression in cancer cells by sponging miR-34, leading to T-cell exhaustion. This circRNA can be found in exosomes from the plasma of lung adenocarcinoma patients and may help in early diagnosis, though more research is needed for its role in melanoma (Xiao et al., 2023). The present state of research on the functions of ncRNAs carried by exosomes in melanoma is summarized in Figure 5.

In cSCC cells, exosomal lncRNA PICSAR promotes resistance to cisplatin through the miRNA-485-5p/REV3L pathway. A study found 25 upregulated and 76 downregulated circRNAs in cSCC patients. Exosomal circ-CYP24A1 is upregulated, and its knockdown limits cell growth and migration while promoting cell death. Rab27a and Rab27b are important proteins for the secretion of exosomes. In melanoma patients, levels of exosomal miRNA-494 are higher. Reducing Rab27a decreases exosome release but increases cellular miRNA-494, leading to less aggressive melanoma cell behavior through increased cell death. This suggests that targeting exosomal miRNA-494 could help treat melanoma. Additionally, cancer-associated fibroblasts (CAFs) aid tumor growth by reshaping the extracellular matrix, and melanoma-derived exosomes can convert normal fibroblasts into CAFs using the lncRNA Gm26809 (Liu and Li, 2022; Wang D. et al., 2020) (see Table 2).

## Medical use of exosomal ncRNAs in melanoma

### Melanoma prognostic indicators

Cancer research is heavily influenced by how genes are expressed and the use of transcriptomic data from high-throughput sequencing technologies like RNA sequencing (RNA-seq), but many studies focus only on genes with high expression levels. This review questions the idea that high gene expression directly causes cancer and stresses the need to understand the difference between correlation and causation. It points out how traditional data analysis can overlook important gene activities in specific cells, a problem being tackled by new single-cell and spatial transcriptomics, though they have their own challenges (Liu H. et al., 2025).

The main tumor markers used for prognosis and follow-up in advanced melanoma are melanoma inhibitory activity (MIA), S100B, and LDH, but they have limited effectiveness. Recently, researchers are looking at exosomes as a new tool for melanoma prognosis. Studies have focused on how exosomal ncRNAs relate to melanoma growth and spread, highlighting their potential for prognosis. MiRNAs are promising for detecting metastatic melanoma and improving prognosis. Specific exosomal miRNAs, like miRNA-532-5p and miRNA-106b, are found in higher levels in patients with metastatic melanoma, indicating their potential for diagnosis and prognosis. Additionally, elevated levels of miR-211-5p in exosomes were linked to responses to therapy. Other factors like MYC, ZIC2, and PPARG may help find new melanoma biomarkers. Exosomes from melanoma cells can enter the body through endothelial cells, with miR-9 promoting blood vessel formation and spread of cancer by activating the JAK-STAT pathway. Exosomal lncRNAs are important markers for diagnosing and predicting outcomes in melanoma. Research shows that lncRNAs are crucial for tumor growth, spreading, and resistance to treatment. BANCR is the first lncRNA linked to BRAF-mutant melanomas and is often overexpressed in melanoma tissues, indicating it may support cancer progression (Ding et al., 2022). Another lncRNA, SPRY4-IT1, is found at higher levels in melanoma cells than in healthy skin cells and is present in various stages of melanoma. HOTAIR is also highlighted as a significant prognostic marker found in melanoma exosomes. Llme23 is a lncRNA that is overexpressed in human melanoma cell lines and could serve as a specific tool for melanoma screening and detection. In a study of 141 patients with stage III lymph node metastases, two lncRNAs, CASC15 and MALAT-1, were found to be independent predictors of disease recurrence and survival. circRNAs have emerged as new tumor markers, notably found in exosomes. Some have important roles in tumor immunity and could serve as prognostic indicators or therapeutic targets. For instance, circ 0008717 has been shown to promote tumor growth. Another study found a correlation between exosomal hsa_circ_0015286 and factors like lymph node metastasis and tumor size. More research is needed to assess the clinical use of exosomal circRNAs in melanoma (Moreno-Traspas et al., 2016; Masrour et al., 2024).

### Drug delivery methods for melanoma targeted therapy

Exosomes are being recognized as a promising way to develop vaccines that stimulate anti-cancer immune responses. They have benefits such as carrying multiple drugs, protecting them, improving cell uptake, being non-toxic, and targeting specific areas. Research shows that an increased level of the STAT3 protein is vital for melanoma growth. Using a special method, STAT3 siRNA was delivered through microneedles into the skin, effectively reducing melanoma size by targeting the STAT3 gene. Additionally, Wee1 siRNA delivered via nanoparticles led to significant inhibition of the Wee1 gene and caused cancer cell death by damaging DNA. Wee1 is a promising inhibitor for addressing DNA damage response in cancer treatment. Researchers are exploring ways to deliver Wee1 more effectively using exosomes. Stremersch et al. showed how they attached siRNA to exosome-like vesicles, highlighting the need for better understanding of how these molecules work. Wang and colleagues created a new formulation of siRNA-loaded DNA with tumor-targeting elements. Additionally, miRNA could help treat melanoma. There are links between miRNA and BRAF-mutated melanoma, but more research is needed to find new miRNAs related to resistance against standard treatments (Dai et al., 2024). Fattore et al. noted that certain miRNAs contribute to resistance against specific cancer drugs, suggesting that therapies targeting these miRNAs might be more effective. Further research is needed to understand how miR-26a works in melanoma and how it might work with other treatments. Jiang et al. found that miR-21 status is an independent factor for prognosis in patients with skin melanoma. Reducing miR-21 using specific methods decreased melanoma growth by encouraging cell death and increased the effectiveness of chemotherapy and radiation. A preclinical therapy targeting miR-32 also reduced MCL-1 levels in melanoma tumors and worked well with the drug vemurafenib. lncRNAs show potential as new drug targets for melanoma. Various RNA-based methods, such as circular RNAs, antisense oligonucleotides, and CRISPR-Cas9, are being used to develop therapies that target lncRNAs. LncRNAs are essential for assessing how effective immunotherapy is, and their interaction with immune checkpoint factors can help predict responses to treatment. For instance, lncRNAs related to PD-L1 can aid cancer cell growth. Reducing these lncRNAs alongside PD-L1 blockage may help suppress tumors. The presence of MELOE-1, a tumor antigen, helps T cells recognize melanoma cells. While immune checkpoint inhibitors have improved survival rates in advanced melanoma, it is still unclear how exosomal circRNAs affect treatment outcomes. Thus, exploring the potential of exosome cargo for innovative therapies in melanoma is crucial (Wang et al., 2010).

### Exosomal ncRNAs in melanoma immunotherapy: mechanisms and clinical advances

Exosomal non-coding RNAs (ncRNAs) have shown significant potential as regulators in melanoma immunotherapy, impacting tumor progression and the effectiveness of immune-based treatments. The mechanisms by which exosomal ncRNAs operate involve modulation of the tumor microenvironment (TME), influencing immune cell behavior, and fostering conditions that may either support or hinder anti-tumor responses. One of the key contributions of exosomal ncRNAs is the polarization of immune cells within the TME. For example, melanoma-derived exosomal miR-125b-5p upregulates specific markers on M1 macrophages, thus promoting an anti-tumor immune response. Conversely, exosomal miR-21 has been observed to facilitate immune evasion by promoting an M2-like macrophage phenotype, resulting in an immunosuppressive environment. Exosomal ncRNAs also significantly affect T cell dynamics (Liu C. et al., 2025). For instance, miR-93-5p can induce CD8^+^ T cell exhaustion through various signaling pathways, leading to diminished T cell responses. Additionally, exosomal miR-23a released from hypoxic tumors can compromise the cytotoxic functions of Natural Killer (NK) cells, further impairing immune surveillance. Beyond their roles in modulating immune responses, exosomal ncRNAs hold clinical significance. They are being explored as potential biomarkers for melanoma diagnosis and prognosis, providing insight into tumor characteristics and monitoring responses to treatment. Furthermore, targeting exosomal ncRNAs presents a novel therapeutic approach, potentially reversing immune dysfunction and improving immunotherapy outcomes (Liu et al., 2018; Yuan et al., 2023). A comprehensive understanding of how exosomal ncRNAs contribute to immunotherapy resistance, particularly through mechanisms like ferroptosis facilitated by miR-21-3p, underscores their importance. Collectively, exosomal ncRNAs represent pivotal players in the evolving landscape of melanoma treatment, serving as both diagnostic indicators and therapeutic avenues. Exosomal ncRNAs have surfaced as valuable biomarkers that can reveal tumor characteristics and aid in monitoring disease progression and therapeutic response. This is vital for tailoring treatment for melanoma patients, as the detection of specific ncRNAs in exosomes signifies inherently unique tumor attributes. Additionally, the article emphasizes therapeutic approaches designed to modify these exosomal ncRNAs to improve immunotherapies (Melnik et al., 2020). By focusing on particular micro-RNAs within exosomes, there is potential to restore immune functions, thus enhancing treatment effectiveness against melanoma. The comprehension of how exosomal ncRNAs play a role in resistance to immunotherapies is also crucial. For instance, exosomal miR-21-3p has been recognized for its ability to promote ferroptosis, a regulated form of cell death, by downregulating specific proteins that safeguard tumor cells. This biological mechanism not only sheds light on the tumor’s adaptive strategies but also paves the way for improving treatment methods, with the goal of overcoming resistance and enhancing survival rates for melanoma patients (Xu et al., 2022).

## Concluding remarks and future perspectives

The findings highlight the importance of non-coding RNAs in skin cancers. Significant advances have been made in diagnosing and treating melanoma, but there is still much to explore regarding earlier and more effective biomarkers. New methods involving ncRNAs are being studied for their predictive and prognostic roles in cancers like breast cancer, prostate cancer, and melanoma. MicroRNAs and lncRNAs are crucial in the development of melanoma and non-melanoma carcinomas by affecting cell growth, movement, and invasion. CircRNAs could serve as new biomarkers for early skin cancer detection. Non-coding RNAs are linked to key cancer characteristics and their misregulation relates to cancer features. They are important in the initial stages of cancer spread, including the EMT process. Understanding how non-coding RNAs influence EMT can lead to new treatments and help identify markers for skin cancers. Further research into these RNAs may also improve cancer treatment strategies. Exosomal ncRNAs are particularly promising as they can deliver drugs or genes effectively, overcoming cancer drug resistance and enhancing chemotherapy effectiveness against tumors. Research shows that engineered exosomes with ncRNAs can significantly improve treatment outcomes. To isolate pure exosomes from various sources, techniques like ultracentrifugation and commercial kits are used, while nanoparticle tracking analysis and Western blot are common for identification. However, these methods have drawbacks, such as high costs, time demands, and risk of contamination, and accurately measuring exosome size and quantity remains challenging. Various cells have been found to generate exosomal ncRNAs, but more research is needed to understand how these are secreted and how they interact with target cells. While exosomal ncRNAs show great potential for clinical use, technical limitations need to be addressed before they can be widely integrated into melanoma treatment.
